# Melanoma-associated B cells are distinct from peripheral blood-derived B cells, and may serve as a source of tumor-targeting antibodies

**DOI:** 10.1186/2051-1426-1-S1-P180

**Published:** 2013-11-07

**Authors:** Daniel Abate-Daga, Theresa A Alexander, Evgeny Arons, Mitchell Ho, Paul F Robbins, Steven A Rosenberg, Richard A Morgan

**Affiliations:** 1 Surgery Branch, Center for Cancer Research, National Cancer Institute, NIH, Bethesda, MD, USA; 2 Laboratory of Molecular Biology, Center for Cancer Research, National Cancer Institute, NIH, Bethesda, MD, USA

## 

The tumor microenvironment contains a complex mix of cell types that can modulate the anti-tumor immune response. In the present study we performed a characterization of tumor-associated B cells, aimed at understanding the biological implications of B cell infiltration in metastases of melanoma patients. We performed a comparative analysis between B cells present in peripheral blood (PBMC) of metastatic melanoma patients and those present in single cell suspensions prepared from metastatic lesions (FrTu) of the same patients. Flow cytometry analysis of these populations showed that the mean B-to-T cell ratio was 5-fold higher in FrTu than in PBMC (p= 0.004, n= 14). Spectratyping of immunoglobulin genes present in FrTu-B revealed a profile that is compatible with an oligoclonal population, as opposed to the polyclonal distribution observed in PBMC-B. Next, we analyzed the expression of 511 genes related to immune cell function in PBMC-B and FrTu-B cells of seven patients. Using a digital platform for direct quantitation of mRNA molecules, we identified forty genes that were differentially expressed (fold change>2, p<0.05), including cytokines (IL6, TNF), death receptors (FAS), differentiation makers (SELL) and transcription factors (BCL6, IKZF1, ETS1, ZEB1, NFKB2), among others. In addition, we analyzed mRNA expression of activation-induced cytidine deaminase (AICDA), an enzyme induced upon B cell activation. We found that AICDA expression was significantly higher in FrTu-B than in PBMC-B cells (p=0.03, n=6). Basal expression of HLA-DR in both FrTu- and PBMC-B cell, as well as induced CD86 expression upon stimulation, suggest that B cells may act as antigen presenting cells and contribute to the initiation of a T cell-based anti-tumor immune response. Our results show that melanoma-associated B cells are distinct from peripheral blood B cells in terms of abundance, clonality and gene expression patterns. Our current efforts are focused on the analysis of FrTu-B cell specificity by lymphocyte display of single chain-variable fragment libraries derived from FrTu-B, with special interest in the identification of antibodies that recognize tumor-specific neoantigens resulting from somatic mutations, for their potential use in personalized chimeric antigen receptor-based adoptive immunotherapies.

